# Respiratory motion artefacts in Gd-EOB-DTPA (Primovist/Eovist) and Gd-DOTA (Dotarem)-enhanced dynamic phase liver MRI after intensified and standard pre-scan patient preparation: A bi-institutional analysis

**DOI:** 10.1371/journal.pone.0230024

**Published:** 2020-03-20

**Authors:** Christian Wybranski, Florian Siedek, Robert Damm, Angelos Gazis, Ortrud Wenzel, Stefan Haneder, Thorsten Persigehl, Susanne Steinhauser, Maciej Pech, Frank Fischbach, Katharina Fischbach

**Affiliations:** 1 Institute of Diagnostic and Interventional Radiology, Faculty of Medicine and University Hospital of Cologne, University of Cologne, Cologne, Germany; 2 Department of Radiology and Nuclear Medicine, University Hospital of Magdeburg, Magdeburg, Germany; 3 Institute of Medical Statistics and Computational Biology, University Hospital of Cologne, Cologne, Germany; Medical University of Vienna, AUSTRIA

## Abstract

**Objective:**

The objective of this study is to evaluate if intensified pre-scan patient preparation (IPPP) that comprises custom-made educational material on dynamic phase imaging and supervised pre-imaging breath-hold training in addition to standard informative conversation with verbal explanation of breath-hold commands (standard pre-scan patient preparation–SPPP) might reduce the incidence of gadoxetate disodium (Gd-EOB-DTPA)-related transient severe respiratory motion (TSM) and severity of respiratory motion (RM) during dynamic phase liver MRI.

**Material and methods:**

In this bi-institutional study 100 and 110 patients who received Gd-EOB-DTPA for dynamic phase liver MRI were allocated to either IPPP or SPPP at site A and B. The control group comprised 202 patients who received gadoterate meglumine (Gd-DOTA) of which each 101 patients were allocated to IPPP or SPPP at site B. RM artefacts were scored retrospectively in dynamic phase images (1: none– 5: extensive) by five and two blinded readers at site A and B, respectively, and in the hepatobiliary phase of the Gd-EOB-DTPA-enhanced scans by two blinded readers at either site.

**Results:**

The incidence of TSM was 15% at site A and 22.7% at site B (p = 0.157). IPPP did not reduce the incidence of TSM in comparison to SPPP: 16.7% vs. 21.6% (p = 0.366). This finding was consistent at site A: 12% vs. 18% (p = 0.401) and site B: 20.6% vs. 25% (p = 0.590). The TSM incidence in patients with IPPP and SPPP did not differ significantly between both sites (p = 0.227; p = 0.390). IPPP did not significantly mitigate RM in comparison to SPPP in any of the Gd-EOB-DTPA-enhanced dynamic phases and the hepatobiliary phase in patients without TSM (all p≥0.072). In the Gd-DOTA control group on the other hand, IPPP significantly mitigated RM in all dynamic phases in comparison to SPPP (all p≤0.031).

**Conclusions:**

We conclude that Gd-EOB-DTPA-related TSM cannot be mitigated by education and training and that Gd-EOB-DTPA-related breath-hold difficulty does not only affect the subgroup of patients with TSM or exclusively the arterial phase as previously proposed.

## Introduction

Respiratory motion (RM) during liver dynamic phase contrast-enhanced Magnetic Resonance Imaging (DCE-MRI) substantially degrades image quality and increases the economic burden for health care systems if examinations need to be repeated. Transient severe respiratory motion (TSM) is a well-known phenomenon after administration of gadoxetate disodium (Gd-EOB-DTPA; Primovist®/Eovist®, Bayer HealthCare Pharmaceuticals) that might impede image interpretation especially of the hepatic arterial phase. The reported incidence of TSM shows a considerable variation of 5–22% between institutions [1–8]. Its pathophysiology is not yet fully understood.

A technical approach to mitigate the effects of Gd-EOB-DTPA-related TSM comprises accelerated MR imaging with short breath-hold times [9–11], multiple arterial phase imaging [12] or free breathing protocols [13,14]. However, these imaging techniques require sophisticated hard- and software, which might not be available at every institution and despite these technological advances, best image quality is achieved in patients without RM during dynamic phase image acquisition. Alternative strategies to reduce the incidence of TSM and severity of RM in the first place are urgently needed. One alternative strategy that has been described previously to minimize TSM was the modification of the injection protocol of Gd-EOB-DTPA. Kim et al. [15] as well as Polanec et al. [16] found a 50% dilution of Gd-EOB-DTPA at an injection rate of 2mL/s [15] or 1mL/s [16] while Davenport et al. [17] found a fixed dose of 10mL instead of 20mL to reduce Gd-EOB-DTPA-related TSM significantly. Another alternative strategy recently described was a modified breathing command that has been advocated to reduce Gd-EOB-DTPA-related TSM [18,19]. The rationale behind this modification was that accustoming patients to the pace and nature of breath-holding would be beneficial to reduce RM in general and consequently also TSM. Another important aspect with regards to Gd-EOB-DTPA-related TSM that has not been evaluated in detail yet is pre-scan patient preparation. Explanation of dynamic phase imaging and breathing commands during informative conversation before image acquisition is clinical standard of care (standard pre-scan patient preparation–SPPP), yet communication about the significance of dynamic phase imaging for diagnosis and the effects of RM might differ between institutions. To the best of our knowledge, supervised pre-imaging breath-hold training is not routinely performed in all institutions. These factors might contribute to the variable incidence of Gd-EOB-DTPA-related TSM.

Hence, the purpose of our bi-institutional study was to investigate if intensified pre-scan patient preparation (IPPP) that focusses on dynamic phase imaging and comprises custom-made educational material and standardized breath-hold training might reduce the incidence of Gd-EOB-DTPA-related TSM and the severity of RM during liver DCE-MRI. The effect of IPPP was crosschecked in patients who received gadoterate meglumine (Gd-DOTA; Dotarem®, Guerbet) for dynamic phase imaging.

## Materials and methods

The ethical commission of the Otto-von-Guericke University and the University Clinic of Magdeburg, Germany, (Approval number: 31/14) and the ethical commission of the University of Cologne, Germany, (Approval number: 18–225) both waived the need for consent as all studies were necessary and medically indicated and our intervention did not influence patient care or patient health while all patient data were also analyzed anonymously. Hereafter, the University Clinic of Magdeburg, Germany, is referred to as site A while the University Clinic of Cologne, Germany, is referred to as site B.

### Standard pre-scan preparation (SPPP)

SPPP was performed consistently at both sites and comprised informative conversation accompanied by standardized informed consent documentation (Thieme Compliance®). All patients were informed about the necessity of breath-holding during dynamic phase imaging, potential sensations associated with contrast agent administration and how to behave at the onset of dyspnea.

### Intensified pre-scan preparation (IPPP)

**Fig 1:**
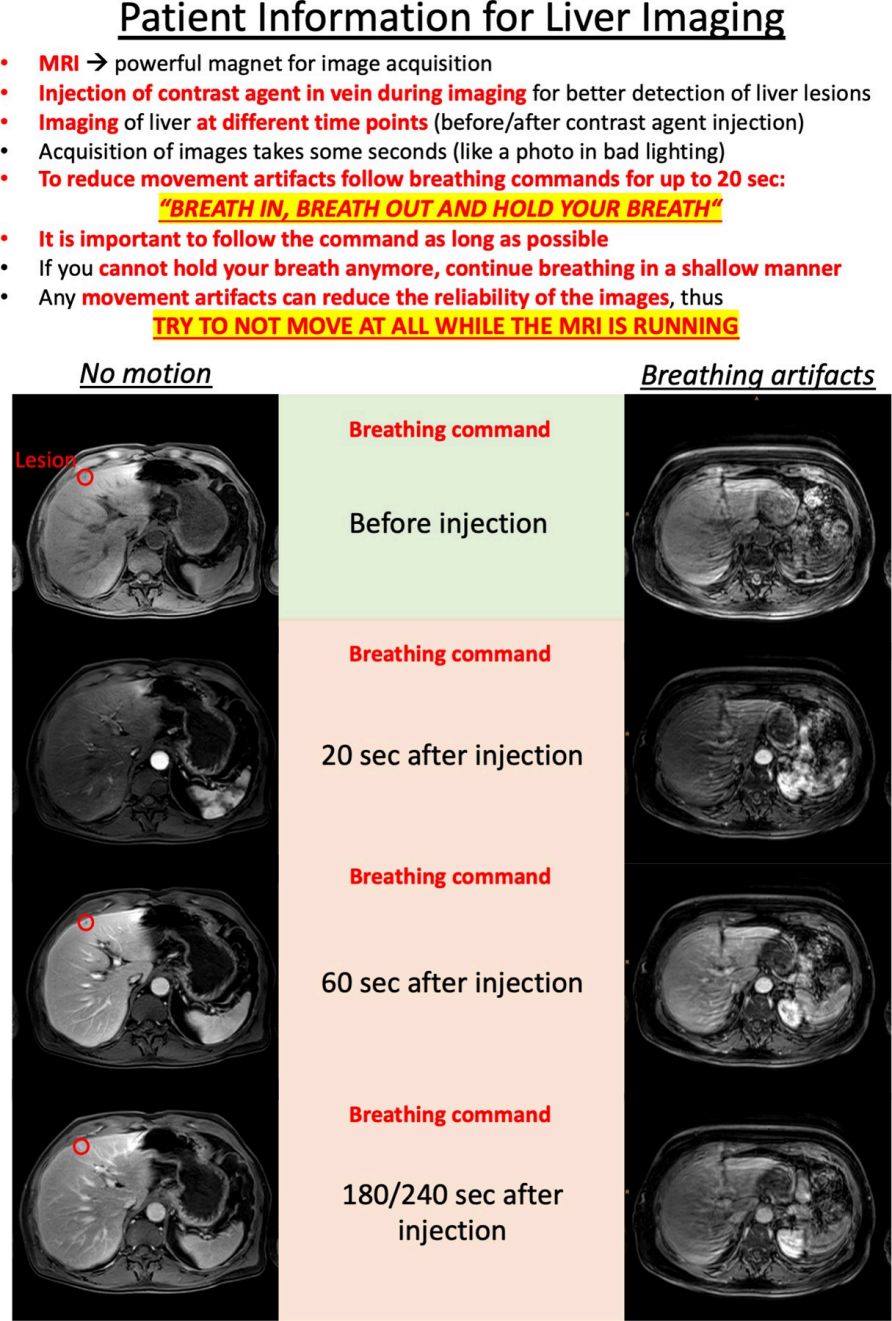
Educational material for intensified pre-scan preparation (IPPP). Educational material employed to illustrate the effects of breathing motion during dynamic phase imaging as part of intensified pre-scan preparation (language has been translated for publication purpose).

IPPP comprised all preparatory steps taken in SPPP. During informative conversation an additional focus was placed on dynamic phase image acquisition, such as the number of acquired phases and diagnostic importance of each phase. Custom-made educational material illustrated the effects of RM during image acquisition (Fig 1). Supervised breath-hold training comprised two 20 s breath-hold cycles measured by means of a stopwatch, which were initiated with the same breath-hold command employed during dynamic phase imaging and patients were instructed to continue shallow and regular breathing at the onset of moderate but still bearable dyspnea.

### Patient allocation to SPPP and IPPP

At site A, one board certified radiologist performed IPPP in 50 consecutive patients scheduled for Gd-EOB-DTPA-enhanced liver MRI in between May–August 2013 without dedicated randomization based on the radiologist’s duty in the MRI unit. Fifty consecutive patients with SPPP within the study interval constituted the control group.

**Fig 2:**
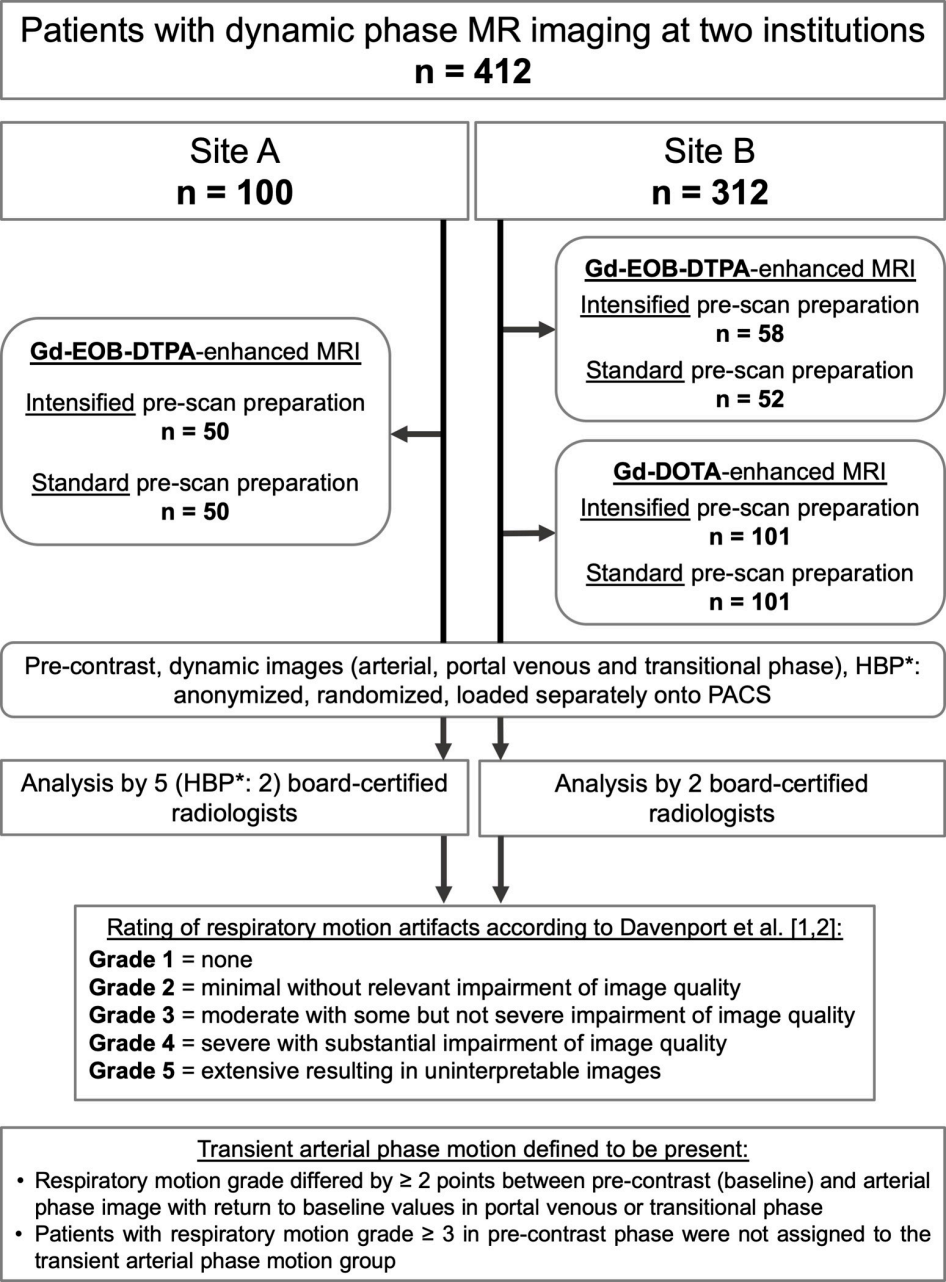
Study flow chart. Patient allocation as well as image analysis protocol for both sites is illustrated. * = HBP: hepatobiliary phase (only applicable in Gd-EOB-DTPA-enhanced scans).

At site B, IPPP and documentation of the accomplished breath-hold duration was performed consecutively in 58 and 101 patients scheduled for Gd-EOB-DTPA- and Gd-DOTA-enhanced dynamic phase imaging by several specialized MR-technicians in between October 2016 –February 2018 without dedicated randomization based on the technicians’ duty in the MRI unit. The technicians who performed IPPP were not involved in the final image acquisition. Fifty-two and 101 consecutive patients scheduled for Gd-EOB-DTPA- and Gd-DOTA-enhanced dynamic phase imaging received SPPP within the study period. The assignment of patients into either group was neither influenced by the investigators nor referring physicians. Patient allocation at both sites is depicted in Fig 2.

### Image acquisition

The detailed technical parameters of T1-weighted (T1w) pre-contrast, dynamic phase imaging and hepatobiliary phase at site A and B are presented in Table 1.

**Table 1:** Technical MRI parameters at sites A and B.

	Site A	Site B	
**Imaging System**	Intera	Ingenia	Ingenia
	(Philips Healthcare)	(Philips Healthcare)	(Philips Healthcare)
**Main magnetic field strength**	1.5 T	1.5 T	3.0 T
**Receiver coil**	Torso 16-channel	Torso 32-channel	Torso 32-channel
**Image sequence**	T1 FFE 3D	T1 FFE 3D	T1 FFE 3D
**Repetition/Echo time (TR/TE; ms)**	3.9/1.84	5.2/2.6	shortest/shortest
**Reconstructed voxel (mm)**	1.0 x 1.0 x 3.0	0.69 x 0.69 x 3.0	1.04 x 1.04 x 2.5
**Sense factor (anterior-posterior/feet-head)**	1.8/1.0	2.0/1.2	2.3/1.3
**Acquisition time for dynamic phases (s)**	14.6	17.0	13.0
**Bolus track**	distal thoracic aorta	distal thoracic aorta	distal thoracic aorta
**Delayed arterial phase (s)**	20	20	20
**Portal venous phase (s)**	60	60	60
**Late venous phase (s)**	180	240	240
Hepatobiliary phase (min)*	20	20	20

Imaging parameters were consistent in pre-contrast and dynamic image phases after contrast agent administration. T = Tesla; FFE = Fast field echo; * = only applicable after Gd-EOB-DTPA administration.

Site A employed exclusively Gd-EOB-DTPA (0.25 mmol/mL) for liver imaging at a fixed dose of 10 milliliters (mL) administered intravenously with an injection rate of 1 mL/s using an automated power injector (Accutron®, Medtronic), followed by a 30 mL saline chaser at the same injection rate. Bolus tracking was used to detect contrast agent arrival in the distal thoracic aorta.

Site B employed Gd-EOB-DTPA (0.25 mmol/mL) or Gd-DOTA (0.5 mmol/mL) for liver imaging based on site specific standard operating procedures (SOPs) and/or the request of the referring physicians. Gd-EOB-DTPA was administered intravenously at a fixed dose of 10 mL with an injection rate of 2 mL/s by means of an automated power injector (Spectris Solaris EP®, Medrad, Bayer Healthcare), followed by a 30 mL saline chaser injected at the same rate. Gd-DOTA was administered weight-adapted with a dose of 0.2 mL/kg with the same injection parameters. Bolus tracking was performed to detect contrast agent arrival in the distal thoracic aorta. Both sites employed an automated breathing command during dynamic phase imaging generated by the imaging system (auto voice): patients were instructed to breathe in and out and stop breathing for image acquisition.

### Image analysis

**Fig 3:**
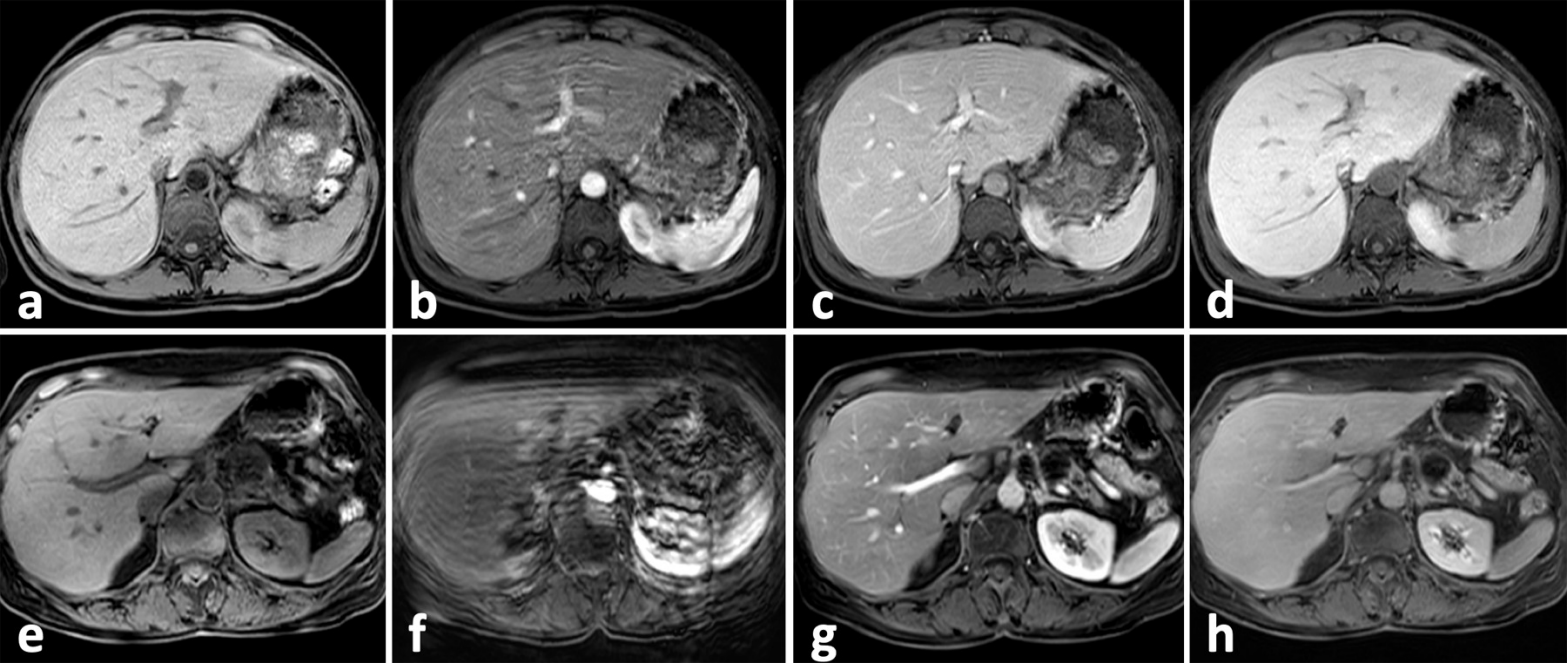
Image examples for TSM after Gd-EOB-DTPA administration. Images (a)-(d): 41-year-old female patient with breast carcinoma and hepatic metastases with TSM after Gd-EOB-DTPA administration. RM scores: 1.0 –pre-contrast; 3.5 –arterial phase; 2.0 –portal venous phase; 1.0 transitional phase. The patient received IPPP prior to imaging. Images (e)-(h): 59-year-old female patient with neuroendocrine pancreatic cancer with TSM after Gd-EOB-DTPA administration RM scores: 1.0 –pre-contrast; 5.0 –arterial phase; 1.0 –portal venous phase; 1.0 transitional phase. The patient did receive SPPP prior to imaging. TSM = Transient severe respiratory motion.

The pre-contrast, arterial, portal venous, transitional and hepatobiliary phase (HBP: only applicable in Gd-EOB-DTPA-enhanced scans) images were anonymized, randomized and loaded separately onto the PACS systems. Five blinded board certified radiologists (HBP: two blinded board certified radiologists) at site A and two blinded board certified radiologists at site B independently analyzed the images for severity of RM. RM was graded according to Davenport et al. [1,2]: Grade 1 = none, Grade 2 = minimal, Grade 3 = moderate with some impairment of image quality, Grade 4 = severe with substantial impairment of image quality, Grade 5 = uninterpretable images (see Fig 3). TSM was diagnosed, if the RM grade differed by ≥ 2 points between pre-contrast and arterial phase image with return to pre-contrast values in portal venous or transitional phase (Fig 3). Patients with RM grade of ≥ 3 in pre-contrast phase were not assigned to the TSM group. The hepatobiliary phase after Gd-EOB-DTPA administration, though not part of the dynamic contrast phases per se, was partly included in the analysis as it might allow a sufficient detection and characterization of focal liver lesions, especially when the arterial phase is uninterpretable due to severe TSM. Accordingly, in addition to the dynamic phases it is also important that the hepatobiliary phase is artifact-free or only has minor artifacts.

### Evaluation of risk factors for Gd-EOB-DTPA-related TSM

Patient characteristics including comorbidities and potential risk factors for TSM were retrieved from the electronic medical record system. Pleural effusion and ascites were measured in the MR images and were scored as moderate (<2 and <5 cm) or severe (>2 and >5 cm). Signs of lung fibrosis or emphysema were evaluated as present or absent in computed tomography studies, whenever available.

### Statistical analysis

Statistical analyses were performed using SPSS Statistics for Windows, version 23.0 (IBM Corp., Armonk, NY). Continuous variables are presented as the median and interquartile range (25^th^ - 75^th^ percentile) and categorical variables as numbers and percentages. RM scores are additionally presented as the mean ± SD. Inter-reader agreement was assessed by calculating the absolute agreement, single measure intra-class correlation coefficient (ICC), applying a two-way random effect model. Pairwise comparisons were performed using the Mann-Whitney *U* test for continuous variables and Pearson's χ2 test or Fisher’s exact test for categorical variables. Fisher’s exact test was performed if at least one cell had an expected count < 5. All reported p-values were calculated based on two-sided test hypotheses and p-values of ≤0.05 were considered statistically significant. As the analyses were regarded as explorative, we did not adjust for multiple testing.

## Results

### Inter-reader agreement for grading of respiratory motion artefacts

The inter-reader agreement for RM grading was excellent (>0.8) or very good (>0.7) at site A and B with ICCs of 0.86 and 0.77 (pre-contrast), 0.92 and 0.89 (arterial), 0.87 and 0.87 (portal venous), 0.84 and 0.73 (transitional phase) as well as 0.86 and 0.75 (hepatobiliary), respectively.

### IPPP and SPPP in Gd-EOB-DTPA-enhanced dynamic phase imaging

**Fig 4:**
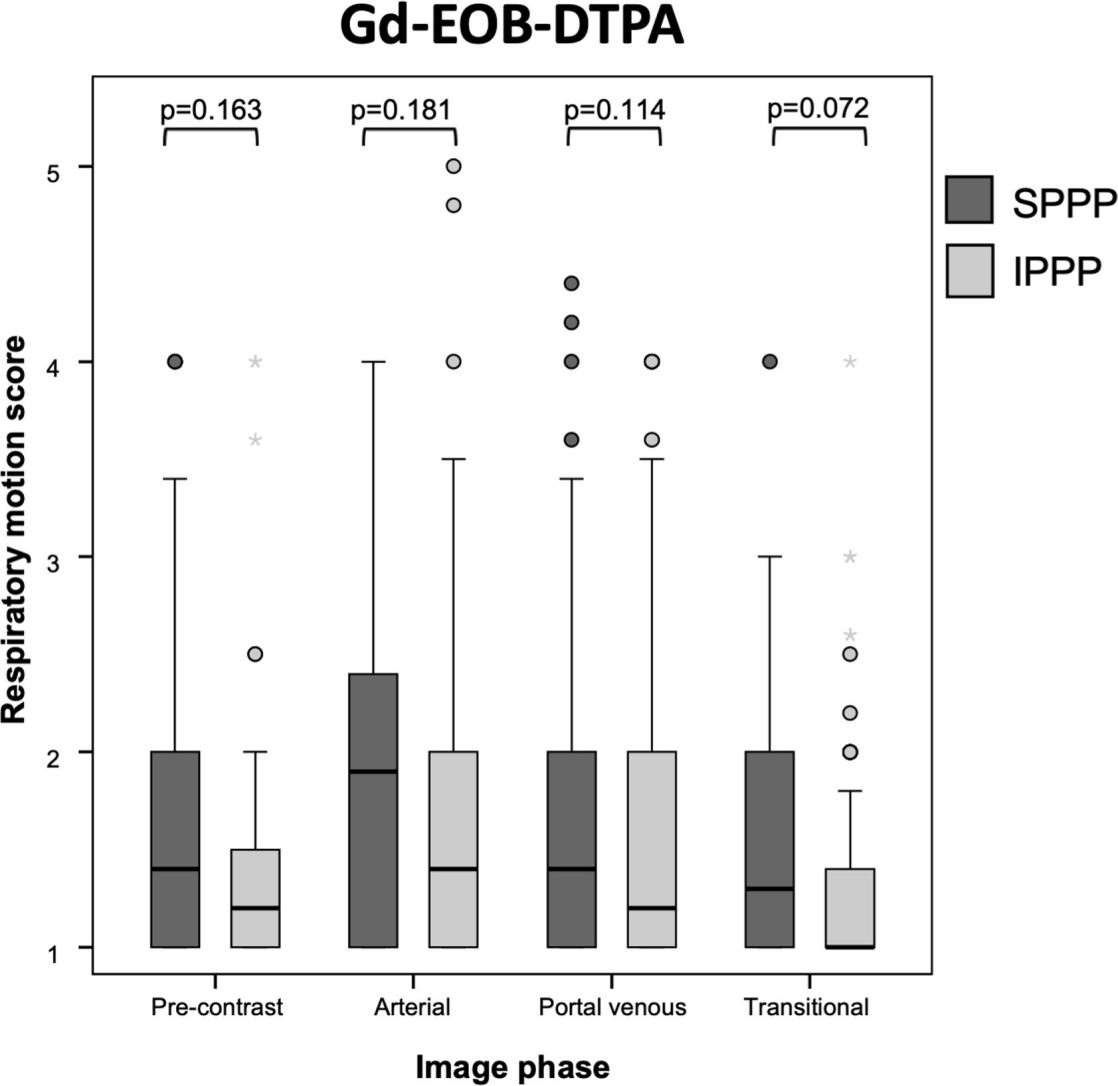
Boxplots with mean respiratory motion (RM) scores of patients without TSM, receiving either SPPP or IPPP prior to administration of Gd-EOB-DTPA. The error bars indicate the minimum and maximum RM score and the boxes depict the interquartile ranges demarcated by median scores. In the Gd-EOB-DTPA group, IPPP did not significantly mitigate RM in arterial (p = 0.181), portal-venous (p = 0.114) and transitional phase (p = 0.072) in comparison to patients who received SPPP.

Patients allocated to SPPP and IPPP did not differ significantly in any of the baseline characteristics (all p≥0.129; Table 2). TSM was observed in 15/100 patients at site A (15.0%) and 25/110 patients at site B (22.7%, p = 0.157). IPPP did not significantly reduce the incidence of TSM in comparison to SPPP: 18/108 patients with IPPP (16.7%) vs. 22/102 patients with SPPP (21.6%; p = 0.366). This finding was consistent at site A: 6/50 patients with IPPP (12%) vs. 9/50 patients with SPPP (18%; p = 0.401); and site B: 12/58 patients with IPPP (20.6%) vs. 13/52 patients with SPPP (25%; p = 0.590). The TSM incidence in patients with IPPP and SPPP did not differ significantly between both sites (site A: p = 0.227; site B: p = 0.390). Out of 170 patients without TSM, 100 patients (58.8%) with prior liver DCE-MRI experience yielded similar RM grades in all dynamic phases compared to the 70 patients (41.2%) who received their first liver DCE-MRI (all p≥0.092). IPPP did not significantly mitigate RM in comparison to SPPP in any dynamic phase in these patients (all p≥0.072; Figs 3 and 4). IPPP also did not significantly mitigate RM in comparison to SPPP in the hepatobiliary phase (p = 0.18).

**Table 2:** Characteristics of patients allocated to either SPPP or IPPP in the Gd-EOB-DTPA and Gd-DOTA group.

		Pre-scan preparation: Gd-EOB-DTPA			Pre-scan preparation: Gd-DOTA			Gd-EOB-DTPA	Gd-DOTA	
		standard	intensified	*p =*	standard	intensified	*p =*	all patients	all patients	*p =*
**Number of patients**		102	108		101	101		210	202	
**Gender**	**female**	48 (47.1)	59 (54.6)	*0.273*	41 (40.6)	29 (28.7)	*0.052*	107 (51)	70 (34.7)	***0.001***
	**male**	54 (52.9)	49 (45.4)		60 (59.4)	72 (71.3)		103 (49)	132 (65.3)	
**Age (years)**	**median (IQR)**	63.4 (52.6–74.0)	60.9 (53.6–71.5)	*0.538*	60.7 (52.5–70.5)	65.0 (53.9–71.5)	*0.225*	61.7 (53.4–73.4)	62.8 (53.6–70.9)	*0.851*
BMI (kg/m^2^) **	**median (IQR)**	25.5 (22.2–31.2)	24.7 (22.5–29.9)	*0.929*	26.8 (24.7–31.1)	26.7 (24.6–31.5)	*0.898*	24.8 (22.4–30.1)	26.7 (24.6–31.4)	***0.013***
**Tumor etiology**	**HCC/CCC**	17 (16.7)	22 (20.4)	*0.700*	25 (24.8)	22 (21.1)	*0.923*	39 (18.6.3)	48 (23.8)	***<0.001***
	**metastasis**	48 (47.1)	52 (48.1)		15 (14.9)	15 (14.9)		100 (47.6)	29 (14.4)	
	**no malignancy**	37 (36.2)	34 (31.5)		61 (60.3)	64 (63.3)		71 (33.8)	125 (61.9)	
**Acquisition time (s)**	**13.0**	34 (33.3)	40 (37.0)	*0.854*	63 (62.4)	68 (67.3)	*0.461*	74 (35.2)	131 (64.9)	***<0.001***
	**14.6**	50 (49.0)	50 (46.3)		.	.		100 (47.6)	0 (0.0)	
	**17.0**	18 (17.6)	18 (16.7)		38 (37.6)	33 (32.7)		36 (17.1)	71 (35.1)	
**Prior MRI**	**yes**	56 (54.9)	63 (58.3)	*0.616*	64 (63.3)	63 (62.3)	*0.884*	119 (56.7)	127 (62.9)	*0.199*
	**median (IQR)**	2 (1–4)	2 (1–5)	*0.233*	2 (1–5)	2 (1–4)	*0.451*	2 (1–5)	2 (1–5)	*0.663*
**Prior TSM**	**yes**	16 (15.7)	26 (24.1)	*0.129*	.	.		42 (20)	.	
**Pleural effusion**	**yes**	11 (10.8)	6 (5.6)	*0.165*	13 (12.9)	9 (8.9)	*0.249*	17 (8.1)	22 (10.9)	*0.333*
	**< 2cm**	9 (8.8)	5 (4.6)	*>0.999*	10 (9.9)	7 (6.9)	*>0.999*	14 (6.6)	17(8.4)	*0.824*
	**> 2cm**	2 (2.0)	1 (0.9)		3 (3.0)	2 (2.0)		3 (1.5)	5 (2.5)	
**Ascites**	**yes**	9 (8.8)	8 (7.4)	*0.707*	18 (17.8)	9 (8.9)	***0.048***	17 (8.1)	27 (13.4)	*0.083*
	**< 5cm**	7 (6.8)	6 (5.5)	*>0.999*	16 (15.8)	7 (6.9)	*0.250*	13 (6.2)	23 (11.4)	*0.585*
	**> 5cm**	2 (2.0)	2 (1.9)		2 (2.0)	2 (2.0)		4 (1.9)	4 (2.0)	
**Cirrhosis**	**yes**	14 (13.7)	19 (17.6)	*0.442*	48 (47.5)	49 (48.5)	*0.500*	33 (15.7)	97 (48.0)	***<0.001***
**Lung fibrosis**	**yes**	3 (3.0)	6 (5.6)	*0.366*	3 (3)	2 (2)	*0.500*	9 (4.2)	5 (2.5)	*0.293*
**CT CM allergy**	**yes**	4 (9.5)	2 (4.2)	*0.412*	1 (1.2)	3 (3.4)	*0.621*	6 (2.8)	4 (2.3)	*0.097*
Allergy general **	**yes**	10/52 (19.2)	12/58 (20.6)	*>0.999*	23 (22.7)	28 (27.7)	*0.859*	22/110 (20.0)	51 (25.2)	*0.476*
Cardiac problems **	**none**	7/52 (13.4)	13/58 (22.4)	*0.502*	22 (21.8)	33 (32.6)	*0.056*	20/110 (18.2)	55 (26.2)	*0.117*

Categories are presented as N (%); SD = Standard deviation; IQR = Interquartile range; Mann-Whitney-U Test was used for quantitative data, all other p-values result from a χ2-test for qualitative data (or Fisher's Test if any cell has an expected cell count less than 5); SPPP = standard pre-scan patient preparation; IPPP = intensified pre-scan patient preparation; HCC/CCC = Hepatocellular/ Cholangiocellular carcinoma; BMI = Body mass index; TSM = transient severe respiratory motion; CT CM = CT contrast media; Allergy general = any allergic disposition to substances or food; Cardiac problems = Hypertension, atrial fibrillation, others;

** = Data was not recorded at site A; significant p-values are depicted in bold.

### Risk factors for Gd-EOB-DTPA-related TSM

Prior episodes of TSM (p = 0.005) and a breath-hold capacity of <17 s during pre-imaging breath-hold training were associated with the occurrence of TSM (p = 0.025; Table 3).

**Table 3:** Risk factors associated with Gd-EOP-DTPA-related TSM.

Risk factors		No TSM	TSM	*p =*
Number of patients		170	40	
**Gender**	**female**	87 (51.2)	20 (50.0)	*>0.999*
	**male**	83 (48.8)	20 (50.0)	
**Age (years)**	**median (IQR)**	61.6 (52.6–73.1)	64.0 (55.9–74.5)	*0.283*
**Tumor etiology**	**HCC/CCC**	29 (17.1)	10 (25.0)	*0.176*
	**metastasis**	80 (47.0)	20 (50.0)	
	**no malignancy**	61 (35.9)	10 (25.0)	
**Prior TSM**	**yes**	27 (15.9)	15 (37.5)	***0.005***
Breath-hold capacity (≥17s) *	**yes**	45/58 (77.6)	13/58 (22.4)	***0.025***
	**median (IQR)**	20 (18.4–20)	20 (16.6–20)	*0.100*
**Pleural effusion**	**yes**	12 (7.1)	5 (12.5)	*0.256*
**Ascites**	**yes**	14 (8.2)	3 (7.5)	*0.878*
**Cirrhosis**	**yes**	28 (16.5)	5 (12.5)	*0.535*
**Lung fibrosis**	**yes**	7 (4.2)	2 (5.1)	*0.797*
**Flow rate (mL/s)**	**1**	85 (50.0)	15 (37.5)	*0.154*
	**2**	85 (50.0)	25 (62.5)	
**Scan time (s)**	**17**	24 (14.1)	12 (30.0)	*0.064*
BMI (kg/m^2^) **		24.9 (22.3–31.0)	24.8 (22.5–28.0)	*0.622*
**CT CM allergy**	**yes**	4 (2.4)	2 (5.0)	*0.595*
Allergy general **	**yes**	18/85 (21.2)	4/25 (16.0)	*0.333*
Cardiac problems **	**yes**	13/85 (15.3)	7/25 (28.0)	*0.100*

Categories are presented as N (%); SD = Standard deviation; IQR = Interquartile range; Mann-Whitney-U Test was used for quantitative data, all other p-values result from χ2-test for qualitative data (or Fisher's Test if any cell has an expected cell count less than 5); HCC/CCC = Hepatocellular/Cholangiocellular carcinoma; TSM = transient severe respiratory motion; CT CM = CT contrast media; BMI = Body mass index; Allergy general = any allergic disposition to substances or food; Cardiac problems = Hypertension, atrial fibrillation, others;

* = Only data from patients at site B with breath-hold training;

** = Data was not recorded at site A; significant p-values are depicted in bold.

### IPPP and SPPP in Gd-DOTA-enhanced dynamic phase imaging

**Fig 5:**
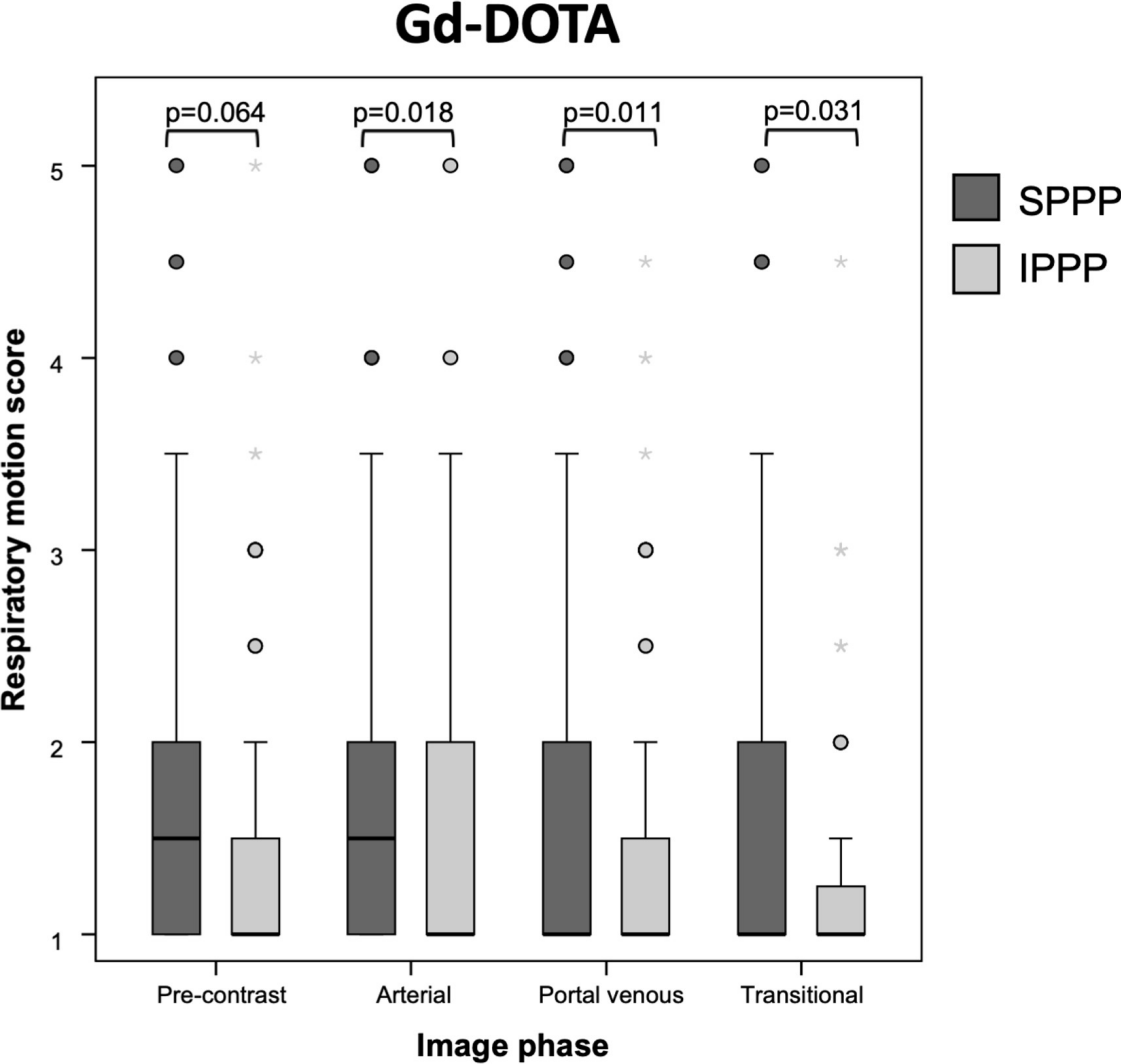
Boxplots with mean respiratory motion (RM) scores of patients without TSM, receiving either SPPP or IPPP prior to administration of Gd-DOTA. The error bars indicate the minimum and maximum RM score and the boxes depict the interquartile ranges demarcated by median scores. In the Gd-DOTA group, IPPP significantly reduced RM in the arterial (p = 0.018), portal-venous (p = 0.011) and transitional phase (p = 0.031).

More patients with moderate ascites were allocated by chance to SPPP (p = 0.048), otherwise baseline characteristics did not differ significantly between patients allocated to SPPP or IPPP (all p≥0.052; Table 2). The Gd-DOTA group comprised more male patients (p = 0.001), with higher mean body mass index (BMI; p = 0.013) and cirrhosis (p<0.001) but less malignant tumors (p<0.001) than the Gd-EOB-DTPA group (Table 2). TSM occurred sporadically in only 4/202 patients (2.0%; p<0.001). One and three patients with TSM were allocated to IPPP and SPPP (p = 0.621). 125/198 patients without TSM (63.1%) have had prior liver DCE-MRI. RM grades were similar in any dynamic phase in patients with and without prior liver DCE-MRI (all p≥0.557). Contrary to the Gd-EOB-DTPA group, IPPP significantly mitigated RM in all dynamic phases in comparison to SPPP (all p≤0.031; Fig 5). Patients who received IPPP in the Gd-DOTA group showed significantly less RM in the arterial, portal-venous and transitional phase (all p≤0.020) than non TSM patients allocated to IPPP in the Gd-EOB-DTPA group, whereas RM was similar in both contrast agent groups in patients who received SPPP (all p≥0.081; Table 4).

**Table 4:** RM scores in patients with SPPP and IPPP in the Gd-EOB-DTPA and Gd-DOTA group.

All patients		RM score	pre-contrast	arterial	portal venous	transitional	hepatobiliary
**Gd-EOB-DTPA**	**n = 210**	Median (IQR)	1.2 (1.0–2.0)	2.0 (1.0–3.0)	1.4 (1.0–2.0)	1.2 (1.0–1.9)	1 (1.0–2.0)
		Mean (SD)	1.5 (0.7)	2.2 (1.1)	1.7 (0.9)	1.4 (0.6)	1.4 (0.7)
		Range	1.0–4.0	1.0–5.0	1.0–4.0	1.0–4.0	1.0–4.0
**Gd-DOTA**	**n = 202**	Median (IQR)	1.0 (1.0–2.0)	1.5 (1.0–2.0)	1.0 (1.0–1.5)	1.0 (1.0–1.5)	
		Mean (SD)	1.5 (0.8)	1.7 (0.9)	1.5 (0.9)	1.4 (0.7)	N/A
		Range	1.0–5.0	1.0–5.0	1.0–5.0	1.0–5.0	
		***p =***	*0.272*	***<0.001***	***<0.001***	***0.007***	***-***
**Patients w/o TSM**							
*SPPP*		**RM score**	**pre-contrast**	**arterial**	**portal venous**	**transitional**	**hepatobiliary**
**Gd-EOB-DTPA**	**n = 80**	Median (IQR)	1.4 (1.0–2.0)	1.9 (1.0–2.4)	1.4 (1.0–2.0)	1.3 (1.0–2.0)	1.0 (1.0–2.0)
		Mean (SD)	1.6 (0.8)	1.9 (0.8)	1.7 (0.8)	1.5 (0.6)	1.4 (0.7)
		Range	1.0–4.0	1.0–4.0	1.0–4.0	1.0–4.0	1.0–4.0
**Gd-DOTA**	**n = 98**	Median (IQR)	1.5 (1.0–2.0)	1.5 (1.0–2.0)	1.0 (1.0–2.0)	1.0 (1.0–1.8)	
		Mean (SD)	1.6 (0.8)	1.8 (0.9)	1.6 (1.0)	1.5 (0.8)	N/A
		Range	1.0–4.0	1.0–5.0	1.0–5.0	1.0–5.0	
		***p =***	*0.775*	*0.159*	*0.081*	*0.197*	*-*
**Patients w/o TSM**							
*IPPP*		**RM score**	**pre-contrast**	**arterial**	**portal venous**	**transitional**	**hepatobiliary**
**Gd-EOB-DTPA**	**n = 90**	Median (IQR)	1.2 (1.0–1.5)	1.4 (1.0–2.0)	1.2 (1.0–2.0)	1.0 (1.0–1.4)	1.0 (1.0–1.3)
		Mean (SD)	1.4 (0.6)	1.7 (0.9)	1.6 (0.9)	1.4 (0.6)	1.3 (0.6)
		Range	1.0–4.0	1.0–5.0	1.0–4.0	1.0–4.0	1.0–4.0
**Gd-DOTA**	**n = 100**	Median (IQR)	1.0 (1.0–1.5)	1.0 (1.0–2.0)	1.0 (1.0–1.5)	1.0 (1.0–1.4)	
		Mean (SD)	1.5 (0.8)	1.5 (0.8)	1.4 (0.8)	1.3 (0.7)	N/A
		Range	1.0–5.0	1.0–5.0	1.0–4.0	1.0–4.5	
		***p =***	*0.231*	***0.007***	***0.001***	***0.020***	***-***

Categories are presented as N (%); SD = Standard deviation; IQR = Interquartile range; RM = respiratory motion; TSM = transient severe respiratory motion; SPPP = standard pre-scan patient preparation; IPPP = intensified pre-scan patient preparation; N/A = not applicable; significant p-values are depicted in bold.

## Discussion

In this bi-institutional study, we strived to investigate if an intensified pre-scan patient preparation (IPPP) could reduce the frequency of Gd-EOB-DTPA-related TSM and the severity of RM during liver DCE-MRI. We crosschecked the effects of IPPP in patients who received Gd-DOTA-enhanced DCE-MRI.

Communication about the significance of dynamic phase imaging for diagnosis and the effects of RM might differ between institutions and this lack of standardization might contribute to the variable incidence of TSM. For that purpose, the bi-institutional approach strengthens the results of this study. Our rationale was to increase patients’ awareness why it is crucial to adhere to breath-hold commands through detailed procedural information analogue to previous studies conducted to reduce unintentional head or limb movement during MRI [20,21]. Supervised breath-hold training in a standardized way aimed to increase patients’ ability to cope with breath-holding, train adequate behavior at the onset of dyspnea and potentially increase breath-hold duration [22].

In our study, the frequency of TSM was lower in the IPPP than in the SPPP group, but without statistical significance. The TSM frequency discovered in our study matched the TSM frequency described previously in the literature [1–8] which corroborates the hypothesis that Gd-EOB-DTPA acts as a chemo-toxic trigger evoking TSM that cannot be willingly mitigated by education and training. Our results differ from the results of Gutzeit et al. [18] and Song et al. [19]. The authors reduced the incidence of TSM from 13% to 0% (4/30 vs. 0/30 patients) [18] and from 14% to 3.8% (14/100 vs. 3/80 patients) [19] by employing a modified breath-hold command with several breathing cycles prior to imaging. We speculate that additional mechanisms of action aside from training and habituation, as proposed by the authors, might have been activated through slow deep breathing, such as optimization of oxygenation [23] or short-term reduction of sympathetic activation and chemo-reflex response [24,25]. Such mechanisms would not have been targeted with our strategy. In our patient cohort, prior episodes of TSM were significantly associated with the occurrence of TSM, consistent with other studies [3,26], whereas other risk factors reported in the literature, such as age [6], gender [6,7,27] or BMI [5,28,29] were not. We identified impaired breath-hold capacity <17 s during breath-hold training as an additional risk factor for TSM. Interestingly, IPPP did not significantly mitigate RM in any of the Gd-EOB-DTPA-enhanced dynamic phases in patients without TSM, whereas it significantly reduced RM in all dynamic phases in patients who received Gd-DOTA. This finding implies that Gd-EOB-DTPA-related breath-hold difficulty does neither affect only the subgroup of patients with obvious TSM nor exclusively the arterial phase, as proposed in previous studies [1,2,30], but that it affects all dynamic phases albeit to a much lesser extent. To the best of our knowledge, our study is the first that used such a study design and yielded these results.

Despite the difficulty to reduce TSM, we want to emphasize that hepatospecific contrast agents with their unique pharmacokinetic properties cannot be replaced and are still urgently needed for liver lesion detection and characterization as well as the determination of liver function. Currently, the most promising strategies to either improve image quality despite TSM or reduce TSM in the first place, as we anticipated, include the dilution of gadoxetic acid [15,16] and new acquisition methods to shorten the acquisition time [12,31], acquire multiple arterial phase images in one single breath-hold [11,12,32] or acquire artifact-free images during free breathing [13,33,34]. The results from new acquisition methods are encouraging but their need for sophisticated hard- and software (parallel imaging techniques: SENSE, GRAPPA, CAIPIRINHA, VIBE, compressed sensing) still constrains their availability.

Our study had some limitations. First, there was no dedicated randomization for IPPP at either site, which might have introduced a selection bias. However, it was performed in consecutive patients based on the staffs’ duty in the MRI unit, which constitutes an element of coincidence. Aside from moderate ascites in the Gd-DOTA group, patient characteristics were similar in all patient groups. Second, there might be a bias by choice of contrast agent at site B, which, however, was based on site specific SOPs and not influenced otherwise. Third, injection rate differed between both sites. However, we found no significant association between injection rate and incidence of TSM, corroborating the results by Ringe et al. [35] but contradicting the results by Kromrey et al [31]. Here, it is important to mention that there is a huge variation and considerable overlap of the reported rates of TSM after different injection rates (1 mL/s: 4.8% to 12.9% [5,26]; 2 mL/s: 7.5% to 21.1% [6,8]). Also, some institutions prefer weight-adapted, others fixed doses of gadoxetic acid making comparisons even more difficult. Fourth, acquisition time for the dynamic phases differed between both sites with a near significant association between scan time and TSM (p = 0.064; Table 3). Fifth, the effect of IPPP was measured only indirectly based on RM image artefacts, which is prone to be biased by subjective interpretation. Although the inter-reader agreement in our study was very good to excellent and matched the results of a recent multi-center trial [36], the assessment of IPPP by dedicated patient questionnaires, respiratory waveform analysis [7,10,14,37,38] or including classification of hyper- and hypovascular liver lesions might have added valuable information and should be addressed in future studies.

## Conclusions

In conclusion, IPPP failed to reduce Gd-EOB-DTPA-related TSM and RM in patients without TSM in comparison to SPPP, corroborating the hypothesis that Gd-EOB-DTPA acts as a chemo-toxic trigger evoking breath-hold difficulties which cannot be mitigated by these measures. Interestingly, IPPP, however, seems to be an effective way to mitigate RM in liver DCE-MRI with extracellular contrast agents such as Gd-DOTA. This suggests that Gd-EOB-DTPA-related breath-hold difficulty does neither affect only the subgroup of patients with TSM nor exclusively the arterial phase as previously proposed but rather all patients and all dynamic phases, albeit to a much lesser extent.

## Supporting information

S1 Table De-identified dataset including scan information, all measured motion scores and information on presence of potential risk factors for TSM. (XLSX)

## References

[1] Davenport MS, Viglianti BL, Al-Hawary MM, Caoili EM, Kaza RK, Liu PSC, et al Comparison of Acute Transient Dyspnea after Intravenous Administration of Gadoxetate Disodium and Gadobenate Dimeglumine: Effect on Arterial Phase Image Quality. Radiology. 2013;266(2):452–461. 10.1148/radiol.12120826 23192781

[2] Davenport MS, Caoili EM, Kaza RK, Hussain HK. Matched within-patient cohort study of transient arterial phase respiratory motion-related artifact in MR imaging of the liver: gadoxetate disodium versus gadobenate dimeglumine. Radiology. 2014;272(1):123–131. 10.1148/radiol.14132269 24617733

[3] Bashir MR, Castelli P, Davenport MS, Larson D, Marin D, Hussain HK, et al Affecting hepatic arterial phase MR imaging with gadoxetate disodium is more common in patients with a prior episode of arterial phase motion associated with gadoxetate disodium. Radiology. 2015;274(1):141–148. 10.1148/radiol.14140386 25162310

[4] Luetkens JA, Kupczyk PA, Doerner J, Fimmers R, Willinek WA, Schild HH, et al Respiratory motion artefacts in dynamic liver MRI: a comparison using gadoxetate disodium and gadobutrol. Eur Radiol. 2015;25(11):3207–3213. 10.1007/s00330-015-3736-x 25903709

[5] Hayashi T, Saitoh S, Tsuji Y, Takahashi J, Tagaya N, Hiramoto M, et al Influence of Gadoxetate Disodium on Oxygen Saturation and Heart Rate during Dynamic Contrast-enhanced MR Imaging. Radiology. 2015;276(3):141646. 10.1148/radiol.2015141646 25811243

[6] Shah MR, Flusberg M, Paroder V, Rozenblit AM, Chernyak V. Transient arterial phase respiratory motion-related artifact in MR imaging of the liver: an analysis of four different gadolinium-based contrast agents. Clin Imaging. 2017;41:23–27. 10.1016/j.clinimag.2016.09.004 27736700

[7] Motosugi U, Bannas P, Bookwalter CA, Sano K, Reeder SB. An Investigation of Transient Severe Motion Related to Gadoxetic Acid–enhanced MR Imaging. Radiology. 2016;279(1):93–102. 10.1148/radiol.2015150642 26473642 PMC5562163

[8] Well L, Rausch VH, Adam G, Henes FO, Bannas P. Transient Severe Motion Artifact Related to Gadoxetate Disodium-Enhanced Liver MRI: Frequency and Risk Evaluation at a German Institution. RoFo. 2017;189(7):651–660. 10.1055/s-0043-102940 28445909

[9] Fahlenkamp UL, Wagner M, Nickel D, Adam U, Krueger K, Taupitz M, et al Novel Dynamic Hepatic Magnetic Resonance Imaging Strategy Using Advanced Parallel Acquisition, Rhythmic Breath-Hold Technique, and Gadoxetate Disodium Enhancement. Invest Radiol. 2016;51(1):33–40. 10.1097/RLI.0000000000000203 26322554

[10] Yoo JL, Lee CH, Park YS, Kim JW, Lee J, Kim KA, et al The short breath-hold technique, controlled aliasing in parallel imaging results in higher acceleration, can be the first step to overcoming a degraded hepatic arterial phase in liver magnetic resonance imaging: A prospective randomized control study. Invest Radiol. 2016;51(7):440–446. 10.1097/RLI.0000000000000249 26807896

[11] Gruber L, Rainer V, Plaikner M, Kremser C, Jaschke W, Henninger B. CAIPIRINHA-Dixon-TWIST (CDT)-VIBE MR imaging of the liver at 3.0T with gadoxetate disodium: a solution for transient arterial-phase respiratory motion-related artifacts? Eur Radiol. 2018;28(5):2013–2021. 10.1007/s00330-017-5210-4 29264636

[12] Pietryga JA, Burke LMB, Marin D, Jaffe TA, Bashir MR. Respiratory Motion Artifact Affecting Hepatic Arterial Phase Imaging with Gadoxetate Disodium: Examination Recovery with a Multiple Arterial Phase Acquisition. Radiology. 2014;271(2):426–434. 10.1148/radiol.13131988 24475864

[13] Yoon JH, Yu MH, Chang W, Park JY, Nickel MD, Son Y, et al Clinical Feasibility of Free-Breathing Dynamic T1-Weighted Imaging with Gadoxetic Acid-Enhanced Liver Magnetic Resonance Imaging Using a Combination of Variable Density Sampling and Compressed Sensing. Invest Radiol. 2017;52(10):596–604. 10.1097/RLI.0000000000000385 28492418

[14] Yoon JH, Lee JM, Yu MH, Hur BY, Grimm R, Block KT, et al Evaluation of Transient Motion during Gadoxetic Acid-Enhanced Multiphasic Liver Magnetic Resonance Imaging Using Free-Breathing Golden-Angle Radial Sparse Parallel Magnetic Resonance Imaging. Invest Radiol. 2018;53(1):52–61. 10.1097/RLI.0000000000000409 28902723 PMC6080614

[15] Kim YK, Lin WC, Sung K, Raman SS, Margolis D, Lim Y, et al Reducing Artifacts during Arterial Phase of Gadoxetate Disodium–enhanced MR Imaging: Dilution Method versus Reduced Injection Rate. Radiology. 2017;283(2):429–437. 10.1148/radiol.2016160241 27977329

[16] Polanec SH, Bickel H, Baltzer PAT, Thurner P, Gittler F, Hodge JC, et al Respiratory motion artifacts during arterial phase imaging with gadoxetic acid: Can the injection protocol minimize this drawback? J Magn Reson Imaging. 2017;46(4):1107–1114. 10.1002/jmri.25657 28181333

[17] Davenport MS, Bashir MR, Pietryga JA, Weber JT, Khalatbari S, Hussain HK. Dose-toxicity relationship of gadoxetate disodium and transient severe respiratory motion artifact. Am J Roentgenol. 2014;203(4):796–802. 25055154 10.2214/AJR.13.11587

[18] Gutzeit A, Matoori S, Froehlich JM, von Weymarn C, Reischauer C, Kolokythas O, et al Reduction in respiratory motion artefacts on gadoxetate-enhanced MRI after training technicians to apply a simple and more patient-adapted breathing command. Eur Radiol. 2016;26(8):2714–2722. 10.1007/s00330-015-4086-4 26573682

[19] Song JS, Choi EJ, Park EH, Lee JH. Comparison of transient severe motion in gadoxetate disodium and gadopentetate dimeglumine-enhanced MRI: effect of modified breath-holding method. Eur Radiol. 2018;28(3):1132–1139. 10.1007/s00330-017-5070-y 28986630

[20] Törnqvist E, Mnsson A, Larsson EM, Hallström I. Impact of extended written information on patient anxiety and image motion artifacts during magnetic resonance imaging. Acta radiol. 2006;47(5):474–480. 10.1080/02841850600690355 16796309

[21] Ali SH, Modic ME, Mahmoud SY, Jones SE. Reducing clinical MRI motion degradation using a prescan patient information pamphlet. Am J Roentgenol. 2013;200(3):630–634. 23436854 10.2214/AJR.12.9015

[22] Parkes MJ. Breath-holding and its breakpoint. Exp Physiol. 2006;91(1):1–15. 10.1113/expphysiol.2005.031625 16272264

[23] Namimoto T, Shimizu K, Nakagawa M, Kikuchi Y, Kidoh M, Oda S, et al Reducing artifacts of gadoxetate disodium-enhanced MRI with oxygen inhalation in patients with prior episode of arterial phase motion: intra-individual comparison. Clin Imaging. 2018;52:11–15. 10.1016/j.clinimag.2018.01.020 29494992

[24] Bernardi L, Gabutti A, Porta C, Spicuzza L. Slow breathing reduces chemoreflex response to hypoxia and hypercapnia, and increases baroreflex sensitivity. J Hypertens. 2001;19(12):2221–2229. 10.1097/00004872-200112000-00016 11725167

[25] Narkiewicz K, Van De Borne P, Montano N, Hering D, Kara T, Somers VK. Sympathetic neural outflow and chemoreflex sensitivity are related to spontaneous breathing rate in normal men. Hypertension. 2006;47(1):51–55. 10.1161/01.HYP.0000197613.47649.02 16344363

[26] Kim SY, Park SH, Wu EH, Wang ZJ, Hope TA, Chang WC, et al Transient respiratory motion artifact during arterial phase MRI with gadoxetate disodium: Risk factor analyses. Am J Roentgenol. 2015;204(6):1220–1227. 26001231 10.2214/AJR.14.13677

[27] Im WH, Song JS, Park EH, Kwak HS. Transient severe motion in the arterial phase during gadoxetate disodium-enhanced MR imaging: evaluation of patients with multiple MR examinations. Abdom Radiol. 2017;42(10):2393–2401. 10.1007/s00261-017-1145-0 28396919

[28] Tanaka M, Kishi Y, Esaki M, Nara S, Miyake M, Hiraoka N, et al Feasibility of Routine Application of Gadoxetic Acid-Enhanced MRI in Combination with Diffusion-Weighted MRI for the Preoperative Evaluation of Colorectal Liver Metastases. Ann Surg Oncol. 2016;23(12):3991–3998. 10.1245/s10434-016-5362-5 27357179

[29] McClellan TR, Motosugi U, Middleton MS, Allen BC, Jaffe TA, Miller CM, et al Intravenous Gadoxetate Disodium Administration Reduces Breath-holding Capacity in the Hepatic Arterial Phase: A Multi-Center Randomized Placebo-controlled Trial. Radiology. 2017;282(2):361–368. 10.1148/radiol.2016160482 27509544 PMC5537625

[30] Furlan A, Close ON, Borhani AA, Wu YH, Heller MT. Respiratory-motion artefacts in liver MRI following injection of gadoxetate disodium and gadobenate dimeglumine: an intra-individual comparative study in cirrhotic patients. Clin Radiol. 2017;72(1):93.e1-93.e6. 10.1016/j.crad.2016.08.005 27633725

[31] Kromrey ML, Hori M, Goshima S, Kozaka K, Hyodo T, Nakamura Y, et al Gadoxetate disodium-related event during image acquisition: a prospective multi-institutional study for better MR practice. Eur Radiol. 2020;30(1):281–290. 10.1007/s00330-019-06358-7 31338655

[32] Grazioli L, Faletti R, Frittoli B, Battisti G, Ambrosini R, Romanini L, et al Evaluation of incidence of acute transient dyspnea and related artifacts after administration of gadoxetate disodium: a prospective observational study. Radiol Med. 2018;123(12):910–917. 10.1007/s11547-018-0927-y 30084108

[33] Weiss J, Notohamiprodjo M, Taron J, Martirosian P, Nickel D, Bamberg F, et al Continuous Hepatic Arterial Multiphase Magnetic Resonance Imaging during Free-Breathing. Invest Radiol. 2018;53(10):596–601. 10.1097/RLI.0000000000000459 29494350

[34] Chandarana H, Feng L, Ream J, Wang A, Babb JS, Block KT, et al Respiratory motion-resolved compressed sensing reconstruction of free-breathing radial acquisition for dynamic liver magnetic resonance imaging. Invest Radiol. 2015;50(11):749–756. 10.1097/RLI.0000000000000179 26146869 PMC4598262

[35] Ringe KI, Von Falck C, Raatschen HJ, Wacker F, Hinrichs J. Evaluation of transient respiratory motion artifact at gadoxetate disodium-enhanced MRI—Influence of different contrast agent application protocols. PLoS One. 2018;13(7):e0200887 10.1371/journal.pone.0200887 30024930 PMC6053213

[36] Ringe KI, Luetkens JA, Fimmers R, Hammerstingl RM, Layer G, Maurer MH, et al Characterization of Severe Arterial Phase Respiratory Motion Artifact on Gadoxetate Disodium-Enhanced MRI—Assessment of Interrater Agreement and Reliability. RoFo. 2018;190(4):341–347. 10.1055/s-0044-100727 29448290

[37] Davenport MS, Malyarenko DI, Pang Y, Hussain HK, Chenevert TL. Effect of gadoxetate disodium on arterial phase respiratory waveforms using a quantitative fast fourier transformation-based analysis. Am J Roentgenol. 2017;208(2):328–336. 27929673 10.2214/AJR.16.16860

[38] Park YS, Lee CH, Yoo JL, Kim IS, Kiefer B, Woo ST, et al Hepatic arterial phase in gadoxetic acid-enhanced liver magnetic resonance imaging: Analysis of respiratory patterns and their effect on image quality. Invest Radiol. 2016;51(2):127–133. 10.1097/RLI.0000000000000211 26418367

